# Choroidal remodeling following different anti-VEGF therapies in neovascular AMD

**DOI:** 10.1038/s41598-024-52315-w

**Published:** 2024-01-22

**Authors:** Giacomo Boscia, Nikolai Pozharitskiy, Maria Oliva Grassi, Enrico Borrelli, Marina D’Addario, Giovanni Alessio, Francesco Boscia, Pasquale Viggiano

**Affiliations:** 1 Department of Translational Biomedicine Neuroscience, University of Bari “Aldo Moro”, Piazza Giulio Cesare, 11, Bari, Italy; 2 Ophthalmology Department, San Raffaele University Hospital, Milan, Italy

**Keywords:** Microbiology, Structural biology, Medical research

## Abstract

The purpose of this study was to investigate choroidal morphology remodeling in AMD-associated type 1 macular neovascularization using two different anti-VEGF drugs. We registered 73 treatment-naïve eyes with a diagnosis of exudative AMD and type 1 MNV. Patients received 3 monthly intravitreal aflibercept (n = 36, aflibercept group [AG]) or brolucizumab (n = 37, brolucizumab group [BG]). Baseline best-corrected visual acuity (BCVA) and anatomical (structural optical coherence tomography) parameters were collected at “T1 control”, before the loading phase (LP) of intravitreal injection, and at “T2 control”, 1 month after the last injection. The main outcomes measured were choroidal vascularity index (CVI), sub-foveal choroidal thickness (SFCT), and central macular thickness (CMT). Our results displayed significant SFCT reduction in both groups between T1 and T2 (*p* < 0.05), We did not find choroidal vascularity modifications (*p* > 0.05) after the loading aflibercept injections. Moreover, only the BG displayed a significant choroidal remodeling (stromal choroidal area [SCA], total choroidal area [TCA] and CVI) at T2 (*p* < 0.05). In particular, a relevant stromal and total choroidal volume reduction was noted, accompanied by an increase in CVI. To conclude, the latter modifications of the choroidal morphology were found significant between two groups (p < 0.05). Our analysis showed a significant impact of brolucizumab on choroidal morphology in eyes affected by type 1 nAMD. This effect was found relevant when compared with aflibercept.

## Introduction

Age-related macular degeneration (AMD) represents the primary cause of irreversible vision loss among people older than 55, with 200,000 new diagnosis per year in the United States^1^. The neovascular form (nAMD) is characterized by the growth of a neovascular network (macular neovascularization) across Bruch’s membrane into the subretinal space. Frequently, resulting in fluid exudation, hemorrhage and hence continuous damage to photoreceptors and retinal pigment epithelium (RPE)^2–5^. Several therapies have been proposed to inhibit the macular neovascularization (MNV)-related exudation^6,7^.

Present day, the principal nAMD therapy is represented by anti-vascular endothelial growth factor (anti-VEGF) injections, which has revolutionized the management of affected patients^8–10^. Different anti-VEGF molecules are available to control exudation and the new vascular network^8,11,12^. Among them, aflibercept (Eylea; Regeneron, Tarrytown, NY, USA, and Bayer HealthCare, Berlin, Germany) has demonstrated robust outcomes in several trials, providing excellent affinity to VEGF^12^.

Recently, the registration of Brolucizumab (Beovu; Novartis, East Hanover, NJ, USA) has expanded treatment options. This antibody fragment is small and light (26 kDa) compared to other anti-VEGF drugs, therefore allowing for higher concentrations during administration^9,13^. Two registration trials (HAWK and HARRIER) reported solid results in maintaining of neovascular exudation^9^.

Through the structural optical coherence tomography (OCT), several authors examined anti-VEGF effects on retinal and choroidal morphology. The choroidal modification is particularly investigated as it is considered the primary layer affected for the neovascularization development^14,15^. During LP of brolucizumab injections, Tamshiro et al.^16^ reported significant anatomic choroidal changes in both the treatment nAMD naïve and switched groups. Likewise, Koizumi et al.^17^ have found SFCT decreased over 12 months with aflibercept injections. Additionally, this choroidal contraction appeared to be related to better visual and anatomical outcomes^17^. Therefore, a careful choroidal inspection may be crucial for the optimal nAMD eyes management. However, the effect of different anti-VEGF drugs on the choroidal vasculature and consequent remodeling remains unclear.

In this observational study, we evaluated modifications of choroidal morphology after loading doses of two different anti-VEGF drugs (aflibercept and brolucizumab) in eyes affected by nAMD naïve and type 1 MNV. We excluded MNV type 2 and type 3 primarily due to their substantial impact on choroidal visualization. The presence of significant fluid, extensive subretinal hyperreflective material (SHRM), and hemorrhages associated with these types significantly interferes with the clarity of choroidal imaging. Moreover, our intention was to ensure a sample of patients with homogeneous anatomical characteristics.

## Results

### Population characteristics

Seventy-three Caucasian patients presenting neovascular AMD and type 1 MNV naïve were included in this study. Among them, 38 were male (52%), and 35 (48%) were female. The mean age was 74.25 ± 9.6 years. The comparison between two groups did not display significantly changes (p ≥ 0.05). The characteristics of this study cohorts are summarized in Table 1.

**Table 1:** The clinical characteristics of subjects included in the analysis.

Variables	Aflibercept group	Brolucizumab group	p value
Number of patients	36	37	> 0.05
Number of eyes	36	37	> 0.05
Age (years)	76.6 ± 5.3	72.89 ± 6.1	> 0.05
Gender (female, %)	17 (48%)	18 (52%)	> 0.05
Pseudophakia, n. of eyes	28	32	> 0.05
Axial length (mm)	23.71 ± 0.71	24.01 ± 0.85	> 0.05

Data are presented as Mean ± SD.

*n* number.

### Choroidal parameters analysis

The SFCT showed significant reduction in both groups at T2 visit (AG: 242.30 ± 101.87 at T1 and 225.92 ± 93.69 at T2; *p* = 0.033; *BG:* 256.36 ± 181.67 at T1 and 235.63 ± 164.78 at T2; *p* = 0.024) (Table 2). However, in the AG none of choroidal parameters (CVI, LCA, TCA and SCA) exhibited significant modifications (*p* > 0.05) after the LP. Conversely, when compared with baseline, patients undergoing the brolucizumab loading presented an increase of the CVI from 0.75 at T1 to 0.79 at T2 (*p* = 0.025). Furthermore, a reduction of both TCA and SCA at T2 was displayed (*p* = 0.021; *p* = 0.029, respectively) as compared to T1*,* while, although not statistically significant, an increase of LCA was observed after brolucizumab LP injections (*p* > 0.05) (Table 2)*.* Importantly, these choroidal remodeling modifications were significant between the two groups (p < 0.05) (Table 3).

**Table 2:** Choroidal analysis of the Aflibercept and Brolucizumab groups.

	Aflibercept group			Brolucizumab group		
	T1	T2	p value	T1	T2	p value
SFCT (μm)	242.30 ± 101.87	225.92 ± 93.69	0.033	256.36 ± 181.67	235.63 ± 164.78	0.024
LCA (mm^2^)	0.7930 ± 0.3212	0.7789 ± 0.2953	0.715	0.8020 ± 0.3742	0.8153 ± 0.3311	0.095
SCA (mm^2^)	0.3177 ± 0.1431	0.2980 ± 0.1165	0.222	0.3101 ± 0.1374	0.2272 ± 0.1026	0.029
TCA (mm^2^)	1.1922 ± 0.5684	1.0583 ± 0.4964	0.566	1.2272 ± 0.5226	0.9217 ± 0.3989	0.021
CVI (%)	0.77 ± 0.27	0.76 ± 0.46	0.568	0,75 ± 0.05	0,79 ± 0.08	0.025
BCVA(logMAR)	0.47 ± 0.12	0.34 ± 0.16	0.016	0.52 ± 0.16	0.39 ± 0.19	0.028
CMT (μm)	333 ± 99.37	237.3 ± 37.65	0.009	354.36 ± 82.77	272.72 ± 58.93	< 0.001

Data are presented as Mean ± SD.

*SFCT* sub-foveal choroidal thickness, *LCA* luminal choroidal area, *SCA* stromal choroidal area, *TCA* total choroidal area, *CVI* choroidal vascularity index, *T1* before loading anti-VEGF therapy, *T2* after loading anti-VEGF therapy.

**Table 3:** Comparison between Aflibercept group vs Brolucizumab group.

	Aflibercept group (n = 36)	Brolucizumab group (n = 36)	p value
SFCT (μm)	− 7.6 ± 5.3	− 10.4 ± 8.9	0.136
LCA (mm^2^)	− 2.4 ± 1.7	4.1 ± 2.9	0.077
SCA (mm^2^)	− 3.5 ± 2.9	− 28.1 ± 16.2	0.004
TCA (mm^2^)	− 10.2 ± 8.4	− 25.6 ± 14.3	0.018
CVI (%)	− 2.1 ± 1.9	7.7 ± 5.2	0.038
BCVA (logMAR)	19.6 ± 17.1	20.2 ± 19.4	0.122
CMT (μm)	− 21.7 ± 20.6	− 22.4 ± 21.8	0.104

Data are delta percentages (Mean ± SD).

*SFCT* sub-foveal choroidal thickness, *LCA* luminal choroidal area, *SCA* stromal choroidal area, *TCA* total choroidal area, *CVI* choroidal vascularity index, *BCVA* best-corrected visual acuity, *CMT* central macular thickness.

### BCVA and CMT after LP

We found statistical BCVA modifications after LP in both groups (p < 0.05) (Table 2). With regard to CMT, in the AG was 333 ± 99.37 μm at T1, and then significantly decreased to 237.3 ± 37.65 μm (*p* = 0.009) at T2. Likewise, in the BG, we observed significant CMT reduction from 354.36 ± 82.77 at baseline to 272.72 ± 58.93 at T2 (*p* < 0.001) (Table 2). Concerning BCVA and CMT analysis, the comparison between two groups did not display significantly changes (p ≥ 0.05) (Table 3).

## Discussion

In this cross-sectional study, we examined the effect of different anti-VEGF drugs on choroidal anatomy in patients affected by neovascular AMD and type 1 MNV naïve. In detail, we found a relevant choroidal vascularity remodeling after brolucizumab loading, while no changes were observed in patients receiving aflibercept injections. Moreover, despite LCA didn’t show any significant modification after the three-monthly injections of brolucizumab, both TCA and SCA showed a significant reduction in these patients. However, a significant sub-foveal choroidal thinning was noted in both groups.

At present, brolucizumab and aflibercept are two of the most effective drugs available for the nAMD treatment^9,12^. As stated above, brolucizumab smaller size allows higher concentrations, permitting a binding capacity to VEGF greater than aflibercept^18^. Despite that, the administration of such drug is still operated with caution due to the multiple reports of intraocular adverse effects occurred after the injections^19^.

Concerning the choroidal structure, several authors have investigated choroidal morphological changes occurring after the intravitreal treatment. Koizumi et al., evaluated a sample of neovascular AMD patients treated with aflibercept over 12 months and showed a significant SFCT decline occurring after the three-monthly injections, which was essentially stable at 12 months^15^. This result is thought to be due to the intravitreal therapy-associated VEGF and nitric oxide suppression, resulting in choroidal vasoconstriction. Likewise, Matsumoto et al. examined the brolucizumab effect on SFCT in 42 eyes with treatment-naïve type 1 MNV. The choroidal thickness decreased by 15.5% after the three loading injections. Accordingly, we found a significant SFCT reduction in both groups, confirming the substantial anti-VEGF impact on the choroidal layer. We added to the literature by assessing choroidal changes as modifications in CVI which depends on the relative modifications of the choroidal vascular and stromal volumes. Analyzing CVI in eyes affected by Polypoidal Choroidal Vasculopathy (PCV), Lee et al.^20^ reported a CVI reduction in affected eyes compared with healthy controls. The authors suggested a choroidal vasculature volume decline or a choroidal stromal volume increase in PCV eyes when compared with controls. Cho et al.^21^ hypothesized that the increased PCV-related choroidal hyperpermeability led to fluid transudation and then increased stromal choroidal volume. In type 1 treatment-naïve MNV in AMD undergoing brolucizumab loading, we found a significant choroidal stromal reduction accompanied by increased CVI at T2. Thus, the CVI increase after brolucizumab injections could be explained by considerable reabsorption of stromal transudation.

Curiously, this significant choroidal remodeling was found only in eyes undergoing brolucizumab therapy. Therefore, aflibercept LP administration may have a different impact on choroidal vasculature as compared with brolucizumab. This finding aligns with earlier research. In a study involving 38 eyes affected by neovascular AMD, Alis et al.^22^ observed a decrease in choroidal thickness but no changes in the choroidal vascularization index. These observations may suggest that brolucizumab is characterized by a greater stromal transudation reabsorption.

This phenomenon had previously been noted in our investigation of patients switched to brolucizumab^23^. Consistent with this observation, we identified an expansion of the choroidal vascular lumen at the expense of the choroidal stroma, leading to a subsequent rise in CVI. This outcome seems more pronounced in treatment-naïve patients. Assuming that, we speculate that the stronger effects reported by brolucizumab in comparison to aflibercept might be due to the smaller size of the molecule, which may better penetrate choroidal tissues whit higher concentrations of the drug in this anatomic space, this leading to a stronger effect of brolucizumab on the chorioretinal morphology. This hypothesis would explain the efficacy of brolucizumab in the treatment of refractory serous pigment epithelial detachment (PED) to previous anti-VEGF^24^. Indeed, the fluid transudation VEGF-related in the stromal area led to the origin of the PED. After brolucizumab loading, an intense contraction of transudate in the stromal space would seem to reduce the hydrostatic pressure and therefore the volume of the PED.

Finally, we observed in both groups a significant BCVA improvement after LP treatment confirming findings from previous studies^25,26^. Consistently, our study cohort showed a significant CMT reduction after both aflibercept and brolucizumab LP injections. Although the influence of central macular thickness on the baseline BCVA is known^27^, its importance during the therapeutic regimen is debatable. Furthermore, we did not observe BCVA and CMT difference between the two groups, this suggesting no different impacts in eyes treated with aflibercept vs. brolucizumab.

The present study has some limitations. First of all, this study included a small number of patients. Another limitation is that the follow-up period was short, and it was not a randomized prospective investigation in which the patients were randomly distributed between a study group and a control group not receiving anti-VEGF drugs. However, also the strengths of our research should be kept in mind. In particular, we included only patients with a naïve nAMD and MNV type 1, as the choroidal morphology may not be altered by previous injections. Furthermore, at the best of our knowledge, no previous paper evaluated CVI in addition to SFCT to compare the effect of different anti-VEGF drugs on choroidal morphology.

In conclusion, our study reports the early choroidal morphological effects occurring in two separate samples of patients after 3 monthly injections of aflibercept or brolucizumab. Our results highlighted the relevant impact on the sub-foveal choroidal thickness of both anti-VEGF drugs, while only the eyes treated with loading brolucizumab exhibited choroidal remodeling. We propose that, after brolucizumab therapy, a greater stromal transudation reabsorption occurs, resulting in stromal and total choroidal volume reduction and therefore an increase of CVI, in comparison with eyes treated with aflibercept. Further prospective, randomized studies are required to confirm our results and to provide a basis for understanding the hemodynamic choroidal changes that occur during anti-VEGF treatment.

## Methods

### Study design

This observational cohort study examined 73 eyes affected by exudative neovascular AMD and type 1 MNV naïve undergoing a loading dose of anti-VEGF treatment (3 monthly anti-VEGF injections) at the retina service of the Department of Translational Biomedicine Neuroscience, University of Bari “Aldo Moro”, Italy between May 2022 and January 2023, 36 treated with aflibercept (2.0 mg/0.05 mL), and 37 with brolucizumab (6 mg/0.05 mL) intravitreal injections for a naïve nAMD. The current research was performed in compliance with the tenets of the Declaration of Helsinki for research involving human subjects and approved by the Ethical Committee of the Department of Translational Biomedicine Neuroscience, University of Bari “Aldo Moro”. An informed consent to participate was signed by each participant before the collection of the following data.

We imaged all patients using XR Avanti spectral domain (SD)-OCT (Optovue, Inc., Fremont, CA, USA). Subjects included were assessed at the following visits: “T1 control”, the day of the first intravitreal injection of the LP, and at “T2 control”, 1 month after the last injection within the loading phase. The T2 visit was carried out following a consistent tracking progression, maintaining the same size and position as the previous evaluation. At each follow-up visit, a complete ophthalmological examination, (BCVA, intraocular pressure measurement and dilated ophthalmoscopy) was performed in enrolled patients.

The inclusion criteria comprised patients affected by neovascular AMD with type 1 MNV. Diagnosis of type 1 neovascularization involved comprehensive assessments, including fluorescein angiography, fundus autofluorescence, indocyanine green angiography, structural OCT, and OCT angiography for all enrolled patients.

Exclusion criteria were: (I) presence of type 2 or type 3 MNV due to the considerable presence of fluid, subretinal hyperreflective material (SHRM) and hemorrhages; (II) presence of opacity lens; (III) uveitis, ocular inflammation or infection, (IV) optic neuropathies including glaucoma, (V) advanced stages of AMD such as disciform scars and geographic atrophy, (VI) previous vitreoretinal surgery or ocular trauma, (VII) a myopia greater than > 3.00 diopters, (VIII) presence of polypoidal choroidal vasculopathy (PCV), (IX) history of any other retinal diseases such as central serous chorioretinopathy, diabetic retinopathy, and retinal dystrophies.

### Outcomes measures

The main outcomes measured were: (i) choroidal vascularity index (CVI); (ii) sub-foveal choroidal thickness (SFCT); (iii) central macular thickness (CFT); and (vi) best-corrected visual acuity (converted into LogMAR scale).

### OCT imaging analysis

Each patient underwent at structural SD-OCT (RTVue XR Avanti) examination using the modality enhanced HD line mode. Acquisitions presenting a poor strength index (SSI < 40), a shadowing effect on the choroid or significant artifacts were excluded.

#### Choroidal vascularity index (CVI)

**Figure 1:**
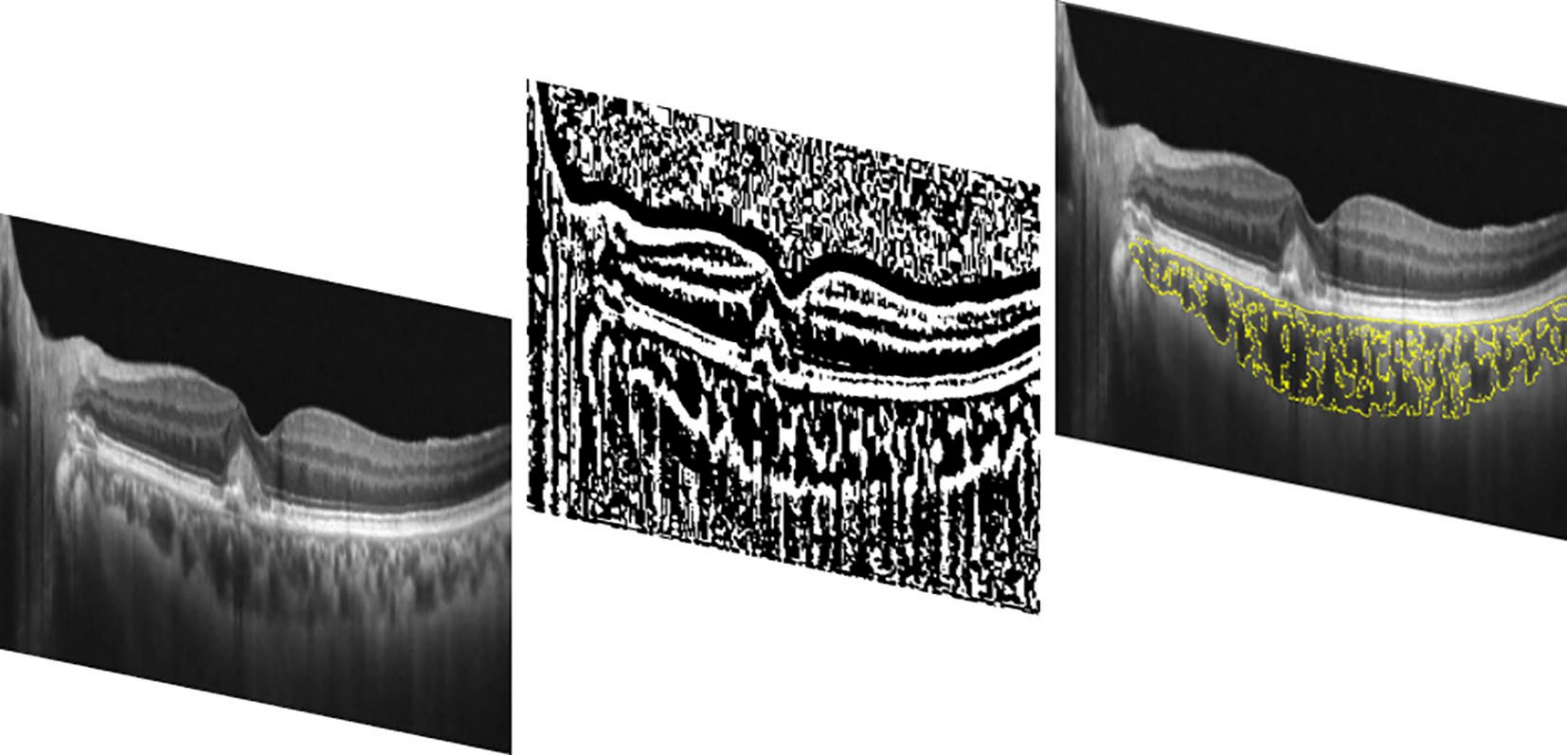
Representation of the methodology used to calculate the choroidal vascularity index. CVI was obtained through a manual identification of the choroid. After conversion into 8 bit, images were binarized through “Niblack’s Auto Local threshold”, dark pixels were defined as the luminal area (LA) and light pixels were defined as stromal area (SA). CVI percentage (%) was calculated by dividing LA for TCA.

Using a previously published methodology^23,28,29^, CVI was obtained through a manual identification of the choroid, defined as the area between the outer border of the RPE and the sclera and therefore known as total choroidal area (TCA). After conversion into 8 bit, images were binarized through “Niblack’s Auto Local threshold”, dark pixels were defined as the luminal area (LA) and light pixels were defined as stromal area (SA) (Fig. 1). CVI percentage (%) was calculated by dividing LA for TCA^30^. Moreover, CVI analysis might be influenced by several factors. For this reason, we applicated a previously reported and validated algorithm^29^.

#### Central macular thickness (CMT)

Using the ETDRS grid system centered on the fovea, CMT was evaluated in the central 1 mm-diameter circle (innermost ring/fovea).

#### Sub-foveal choroidal thickness

SFCT was obtained with the caliper function of structural OCT. The SFCT was measured manually with the caliper from Bruch’s membrane to the sclera-choroidal junction perpendicularly in the center of the fovea by two separate operators (G.B. and P.V.)^31^. Interobserver agreement (average) was found to be excellent in the SFCT assessment (0.90 (confidence interval, 0.86–0.93).

**Figure 2:**
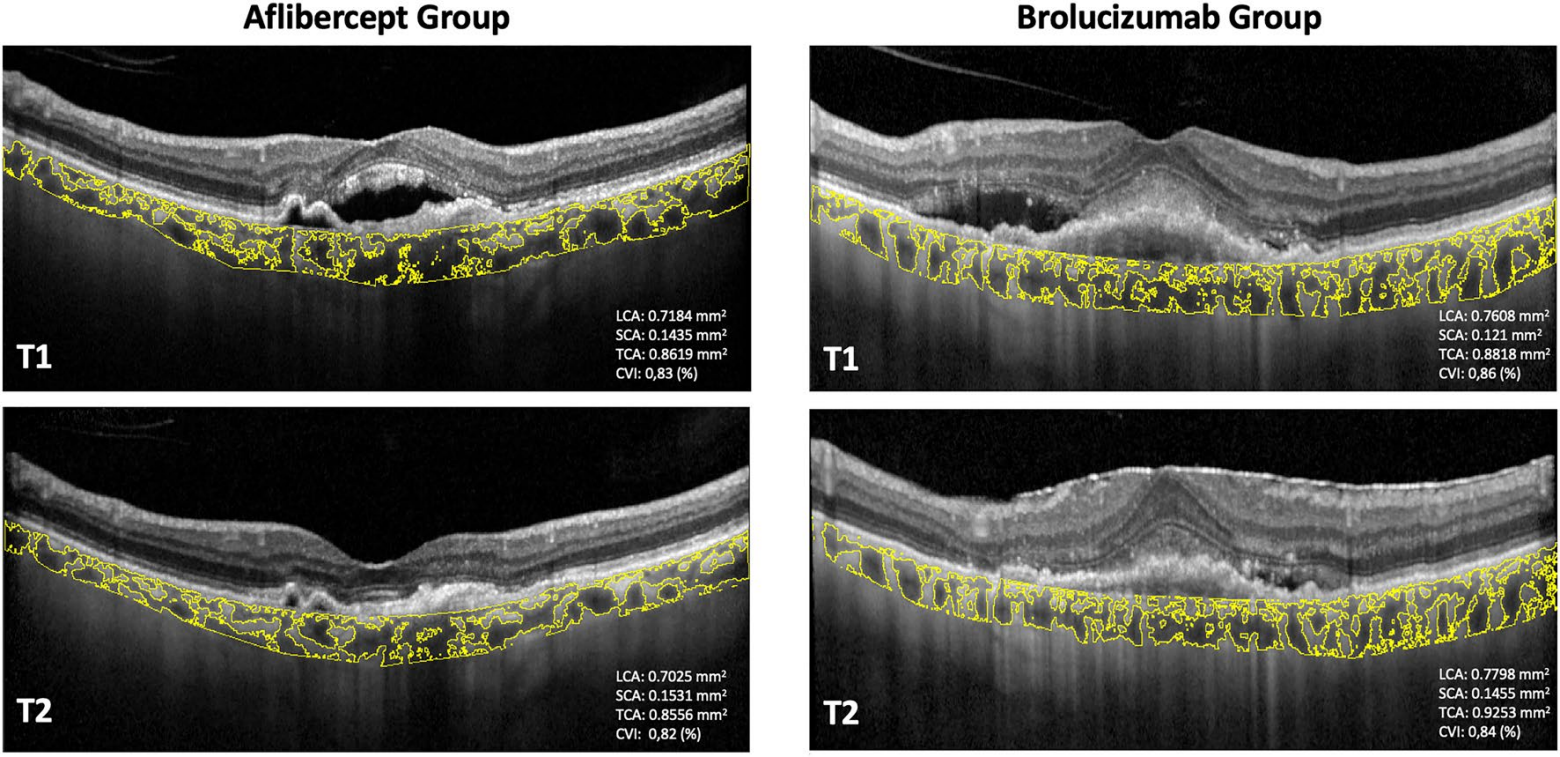
The figure shows CVI changes before and after the loading phase of various anti-VEGF treatments. “T1 control” aligns with the day of the initial intravitreal injection of the LP, while “T2 control” corresponds to one month after the final injection during the loading phase. The T2 visit adhered to a systematic tracking protocol, maintaining consistent size and positioning for comparison with the preceding assessment.

CMT, SFCT, and CVI were examined at each visit (T1 and T2) (Fig. 2).

### Statistics analysis

Statistical calculations were performed using Statistical Package for Social Sciences (version 25.0; SPSS Inc., Chicago, IL). To evaluate the normal distribution of data a Shapiro–Wilk test was determined for all variables. All quantitative variables were reported as mean ± standard deviation (SD). Pairwise comparisons were performed with a paired t-test to compare CVI, SFCT, CMT, and BCVA between follow-up examinations within each group. Friedman non parametric test was employed to compare delta changes between groups. The level of statistical significance was defined as p < 0.05.

## Data Availability

The datasets used and/or analyzed during the current study available from the corresponding author on reasonable request.
